# AFM Characterization of Temperature Effect on the SU-8 Mechanical and Tribological Properties

**DOI:** 10.3390/polym14051009

**Published:** 2022-03-02

**Authors:** Marius Pustan, Corina Birleanu, Rodica Voicu, Raluca Muller

**Affiliations:** 1 Micro & Nano Systems Laboratory, Department of Mechanical Systems Engineering, Technical University of Cluj-Napoca, 103-105 Muncii Street, 400641 Cluj-Napoca, Romania; Marius.Pustan@omt.utcluj.ro; 2 Modeling, Simulation and CAD Laboratory, National Institute for Research and Development in Microtechnologies—IMT Bucharest, 126A Erou Iancu Nicolae Street, 077190 Bucharest, Romania; rodica.voicu@imt.ro (R.V.); raluca.muller@imt.ro (R.M.)

**Keywords:** SU-8, AFM, modulus of elasticity, hardness, adhesion, friction

## Abstract

This study presents the effect of temperature on the mechanical and tribological properties of SU-8 polymer. The temperature of investigated samples increasing during testing and the variation of mechanical and tribological properties were monitored. The samples for tests were SU-8 hard baked at different temperatures. The hard bake temperature changes the mechanical and tribological properties of polymers. The aim of this research work is the reliability design improvement of SU-8 microstructures from electro-thermally actuated devices where a thermal gradient produces the softening and modification of SU-8 behavior. As a function of the hard baked temperature, different mechanical and tribological properties were experimentally determined using the atomic force microscopy (AFM) technique. The mechanical properties of interest are the modulus of elasticity and hardness. The investigated tribological properties involve the adhesion and friction forces. The modulus of elasticity and hardness decrease if the operating temperature increases based on the thermal relaxation of material and their viscoelastic behavior. The adhesion force between AFM tip and investigated samples increases if the operating temperature increases, respectively. The same evolution was experimentally observed in the case of friction force. Moreover, for the same testing temperature, the modulus of elasticity and hardness increase, and the adhesion and friction forces decrease if the SU-8 is hard baked at high temperature.

## 1. Introduction

The SU-8 is an epoxy-based negative photoresist material that was first developed and patented by IBM in 1989 [1,2]. This material has adequate mechanical properties that make it capable of flexible structures manufacturing or as a support for other components. The SU-8 is directly used as a polymeric material to produce mechanical components in the area of Microelectromechanical Systems (MEMS) [3,4]. This polymer exhibits good biocompatibility and has been successfully implemented in Bio-MEMS [5,6].

Additionally, this material is insoluble in water and after polymerization has good chemical durability [3]. The SU-8 allows obtaining surfaces with good roughness, which is a basic need for reliability design of MEMS structures with adequate mechanical and tribological characteristics [3].

First, the SU-8 was developed as a thick-film resist for electroplating in the LIGA process, but it soon became a popular material in other areas of microfabrication including microfluidic, biological, and optical MEMS applications [7].

The fabrication of flexible MEMS structures from SU-8 material shows some advantages over the silicon MEMS technology [8]. For example, the voltage required to operate an electrostatic actuator fabricated from SU-8 can be reduced due to its lower modulus of elasticity that provides smaller mechanical stiffness of structure [9]. In some applications, the MEMS structures are multilayer fabricated using the SU-8 polymer as the structural layer close to the other metal layer as gold or aluminum [9]. The reliable operation of such MEMS structures strongly depends on the tribological and mechanical properties of SU-8 layer and its behavior under different operating conditions including temperature, humidity, and the medium pressure. The operating temperature has influence on the mechanical behaviors of materials as it was already demonstrated based on the other experimental tests including tensile analysis [10].

The tribological and mechanical properties of SU-8 are very sensitive to the processing conditions including the hard bake temperature [2]. Unfortunately, processing uniformity and reproducibility are critical for large-scale industrial manufacturing [3]. In this context, the characterization of the material itself became a vital need to obtain the reliable components reproducibly.

The data of mechanical and tribological properties of SU-8 published in the literature vary substantially as a function of the processing conditions, testing method and the operating parameters [10,11,12]. Different experimental methods have been used to determine the modulus of elasticity of this material. The experimental results of modulus of elasticity obtained by nanoindentation vary between 3.5 GPa and 7.5 GPa as a function of the processing conditions and the testing method [13]. For the SU-8 thin films, nanoindentation testing method is successfully used to characterize the mechanical properties where the modulus of elasticity and hardness can be determined based on the load-indentation depth experimental curve [13,14]. These mechanical parameters depend on the hard bake temperature and are strongly influenced by the operating conditions. Advantages of SU-8 for MEMS application are also highlighted by the fact that it can be used at high temperature environment [10]. Therefore, this material characterization at elevated temperature is essential for the reliability design of MEMS structures [10].

In the analysis of the mechanical properties of SU-8 by using the nanoindentation method, additional difficulties are caused by viscoelastic behavior, especially if the components are operating at high temperature [12]. Polymers show different behaviors when indentation is made in various contact conditions. For this reason, viscoelastic behavior of this material depends on the contact geometry and the indentation depth, loads and environmental conditions [12].

The experimental values of hardness and modulus of elasticity of samples were analyzed in this research work based on the load-displacement curve using the Oliver and Pharr method. Since 1992, this method proposed by Oliver and Pharr has been established as the standard procedure for determining the hardness and elastic modulus from the indentation load-displacement data during one cycle of loading/unloading of bulk materials [15]. However, the Oliver and Pharr method is frequently used by researchers to interpret the indentations performed on thin films to obtain the film properties regardless of the substrate effect on the measurements [13,15]. Although it was originally intended for application with sharp, geometrically self-similar indenters like a Berkovich triangular pyramid.

The objectives of this paper are focused on the influence of temperature on the mechanical and tribological advantages of SU-8 and to highlight the experimental results to obtain quantitative analyses, the elastic/plastic response and the nanotribological behavior. The slight fluctuation of the results for common SU-8 materials is most likely due to the methods used to measure their properties. The experimental characterization of mechanical and tribological properties of SU-8 processed at different hard bake temperature were performed using the atomic force microscopy (AFM) technique. The relationship between the material properties and processing conditions can be used to optimize its application for the reliability design of MEMS. The tribological behavior is discussed in terms of the adhesion and friction forces. In order to investigate the temperature effect on mechanical and tribological properties of this material, the experimental tests were performed under different testing temperature.

The atomic force microscopy is a powerful characterization tool for the polymer science [16,17,18,19]. In the AFM technique, a sharp tip attached to a cantilever is moved across the sample surface to measure the surface morphology on the atomic scale and the friction during scanning. The force between the tip and sample is monitored by the deflection of AFM cantilever. The AFM cantilever also can be vertically monitored for the spectroscopy-in-point analysis and nanoindentation. Spectroscopy-in-point is used for adhesion and stiffness measurements of flexible microcomponents under a controlled force [9,20,21]. In the non-contact mode of AFM, a tip attached to the cantilever is hovering very close to the surface, making it possible to measure the polymeric samples without surface alteration. This technique has been successfully applied for the characterization of multilayer MEMS cantilever fabricated in SU-8 structure [9] and recently for the investigation of a new Poly(3-hydroxybutyrate) material [19].

The scope of experimental analysis is the reliability design improvement of MEMS with integrated SU-8 polymer as the main structural layer. Recent research works developed by authors involved polymeric microgrippers design, their fabrication, and characterization [22,23]. Two-type V-shaped electro-thermally microgrippers were designed using the principle of electrically driven thermal actuation and the V-shaped configurations [22]. The other polymeric microgripper based on SU-8 with a double in-plane action of the arms and a large displacement also had been developed [21]. This microgripper is electro-thermally actuated using three pairs of chevron actuators. The temperature generated in the gripper structure has influence on the SU-8 material behavior with effect on the stiffness and the clamping force of the gripper jaws.

The aim of the mechanical tests was to determine the temperature influence on modulus of elasticity and hardness. The other experimental tests were performed to analyze the temperature effect on the adhesion and friction forces.

## 2. Materials and Methods

### 2.1. Materials

The processing procedure includes spin coating, soft-baking, UV exposure, post-exposure baking and hard-baking. Standard silicon wafers of n-type have been cleaned and used as the substrate for the experimental samples. The SU-8 2015 polymers (Micro-Chem, Newton, Massachusetts) were spined and samples with a thickness of 10µm had been obtained. After, these samples were selected as the testing materials. The SU-8 polymer is an epoxy biocompatible negative photoresist, and it was used in the last period for micro- and nano-fabrication of MEMS devices such as microgrippers for handling and manipulation cells and bio-particles [22,23,24] or lab-on-a-chip systems [8,25].

In order to investigate the processing conditions effects on the material properties, the SU-8 were hard baked at different temperatures to complete the crosslinking of the polymer. Four samples of SU-8 were selected for experimental characterizations with the hard bake temperature of 125 °C, 165 °C, 195 °C and 215 °C. These samples were experimentally investigated to determine their mechanical and tribological behavior variation under different operating temperatures.

Approximatively, Glass Transition Temperature (Tg) of SU-8 is 210 °C. For this reason, four temperatures of investigated samples were selected in order to study the behavior of the polymer before Tg and near/after Tg, keeping in mind the possible applications of this material and the effect of the hard bake temperature step.

### 2.2. Methods

The experimental tests presented in this paper were performed using the AFM XE-70 (manufactured by the Park System Co., Suwon, South Korea) and a thermal controlled stage. The tests were repeated at least 4 times for each analysis and the average results are further presented and discussed.

The testing procedure involves: (i) the analysis of the surface roughness as a function of the processing temperature; (ii) nanoindentation tests for modulus of elasticity and hardness measurements as a function of the processing and operating temperatures; (iii) determination of the adhesion force between the AFM tip and investigated samples for different temperatures; (iv) analysis of the temperature effect on the friction force between the AFM tip and the investigated samples.

The AFM probe used in the surface characterizations is the PPP-NCHR probe with a thickness of 4 µm, a width of 30 µm, a length of 125 µm, a force constant of 42 N/m and a 330 kHz the resonant frequency. This AFM probe has high operation stability and a fast-scanning ability. The scan rate during measurement was selected of 0.75 Hz, the set-point of 2 nm and 20 nm the amplitude of oscillations. The scanning area was 1 µm × 1 µm (51 pixels × 51 pixels).

The AFM probe chosen for the nanoindentation tests was TD 21464 with a Berkovich—shaped tip and a force constant of 156 N/m. Each sample was indented 4 times (in a 2 × 2 matrix) with a force of 50 µN and the average results were considered and presented in this paper. The tests were performed at different operating temperatures: 20 °C, 40 °C, 60 °C, 80 °C and 100 °C.

The AFM probe used in the adhesion analysis was a PPP-NCHR probe with a force constant of 42 N/m and the tip radius of 7 nm (the same probe used in the surfaces characterization). The adhesion tests were repeated 4 times (in a 2 × 2 matrix) for each sample and the average values were considered.

In order to avoid the scratch of investigated polymers during friction analysis, an AFM probe type PPP-LFMR optimized for high sensitivity to lateral force was used. The characteristics of AFM probe are: the force constant of 0.2 N/m, length 225 μm, width 48 μm, thickness 1 μm, the tip height 15 μm.

## 3. Results

### 3.1. Surfaces Topography

The surface parameters have an important influence on the adhesion and friction effects from MEMS devices with movable components.

In order to determine the effect of hard bake temperature on the surface parameters of investigated polymers, the surfaces scanning was carried out by using the non-contact mode of AFM (NC-AFM). The NC-AFM mode measures the surfaces topography based on an attractive atomic force in the relatively larger distance between the AFM tip and the samples. This method is selected to avoid the scratch and deterioration of the polymeric samples.

**Figure 1:**
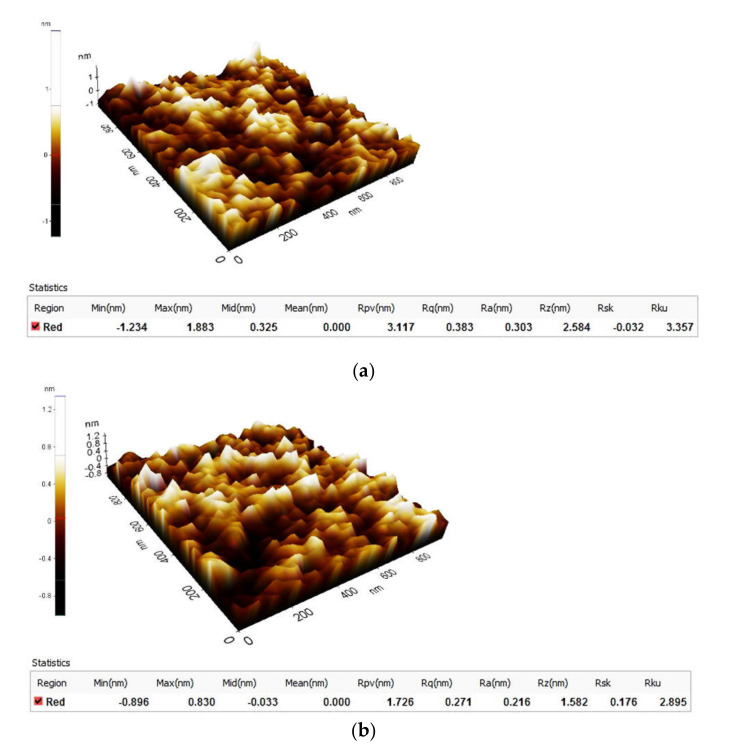
Topography (3D image) and the roughness parameters of: (**a**) SU-8 hard baked at 125 °C; (**b**) SU-8 hard baked at 215 °C.

The hard bake temperature of SU-8 changes the roughness parameters of surfaces. Figure 1 presents the roughness parameters of investigated samples hard baked at 125 °C (Figure 1a) and at 215 °C (Figure 1b). The roughness parameters of surfaces are changed if the hard bake temperature is modified.

The arithmetical mean roughness value (Ra) is one of the most effective surface roughness commonly adopted in the general engineering practice. On the scanning size of 1µm × 1µm (51 pixels × 51 pixels), the measured Ra roughness is 0.303 nm for SU-8 hard baked at 125 °C, it decreases to 0.296 nm for 165 °C the hard bake temperature, 0.268 nm in the case of 195 °C the hard bake temperature and 0.216 nm for the polymer hard baked at 215 °C. At nanoscale, some researchers suggest the root mean square roughness (Rq) as a useful parameter for the analysis of the polymer surfaces [26].

The SU-8 is a thermal resin and as such its properties continue to change when exposed to higher temperatures. We consider that the roughness decreases based on the thermo-chemical transformation of the polymer and the cross-link realizations during the hard bake process. It is well known that the hard bake step is useful for annealing any surface cracks that may be evident after development.

### 3.2. Temperature Effect on Modulus of Elasticity and Hardness

The mechanical properties such as modulus of elasticity and hardness of SU-8 are influenced by the hard bake temperature and the operating conditions. These mechanical properties were investigated based on the nanoindentation module of AFM.

The temperature influence on hardness and modulus of elasticity of investigated samples was experimentally monitored. As the testing temperature increases, the mechanical properties are changed. This modification is based on materials thermal relaxation and their viscoelastic behavior [12].

**Figure 2:**
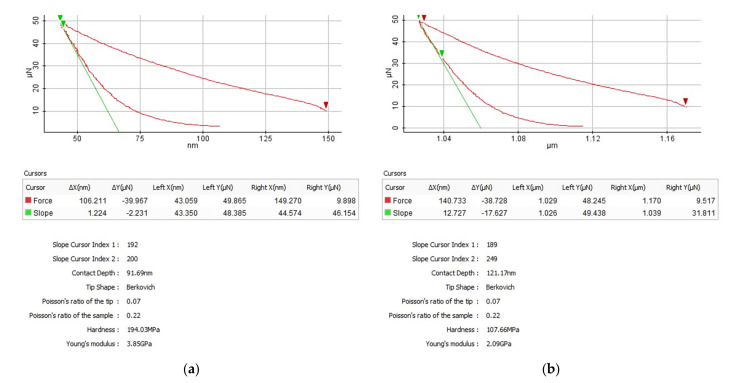
Nanoindentation of SU-8 hard baked at 125 °C, tested at 20 °C (**a**) and 100 °C (**b**).

Figure 2 presents the temperature effect on hardness and modulus of elasticity of SU-8 hard baked at 125 °C. Under the same indentation force, the contact depth increases from 91.69 nm (Figure 2a) to 121.17 nm (Figure 2b) if the testing temperature is increased from 20 °C to 100°C. This effect has influence on the hardness and modulus of elasticity as shown in Figure 2. In the case of SU-8 hard baked at 125 °C, the hardness decreases from 194.03 MPa to 107.66 MPa and the modulus of elasticity from 3.85 GPa to 2.09 GPa if the testing temperature increases from 20 °C to 100 °C. The same influence was observed for all investigated samples.

**Figure 3:**
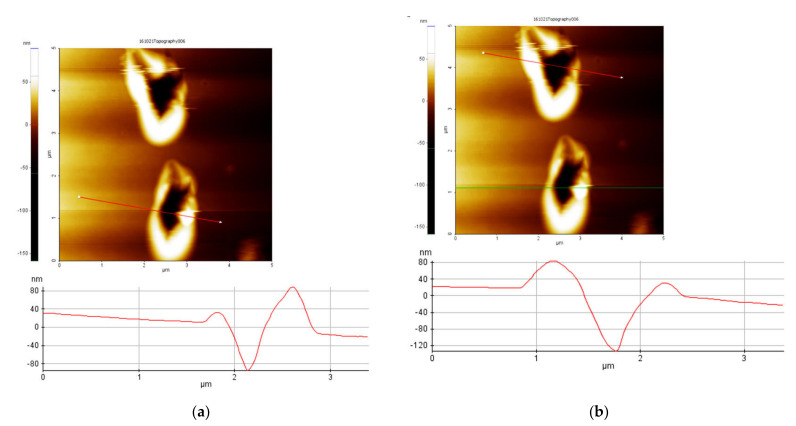
Nanoindentation depths of SU-8 hard baked at 125 °C, tested at 20 °C (**a**) and 100 °C (**b**).

In order, to visualize the indentation depths, the samples surfaces were scanned using the non-contact operating mode of AFM. Figure 3 presents the indentation depth of SU-8 hard baked at 125 °C and tested at 20 °C (Figure 3a) and 100 °C (Figure 3b). The indentation depths correspond with those provided by the load-displacement experimental curves (Figure 2). Under the same indentation force, the indentation depth increases if the testing temperature increasing, respectively. The substrate influence on the modulus of elasticity and hardness is neglected because the indentation depth is much smaller compared with the samples thickness (10 µm). Generally, in the nanoindentation analysis, it is accepted that, if the indentation depth is smaller than 10% of the film thickness, the influence of the substrate can be neglected [27,28].

The hard bake conditions such as temperature severely affect the material properties of SU-8 polymers.

**Figure 4:**
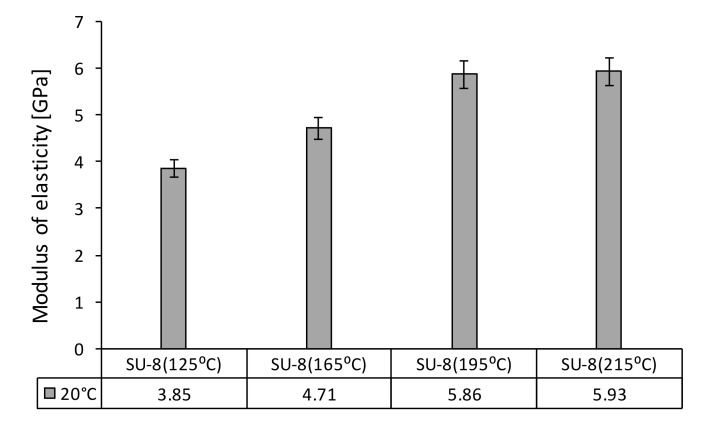
Modulus of elasticity of SU-8 hard baked at 125 °C, 165 °C, 195 °C, 215 °C tested at 20 °C.

**Figure 5:**
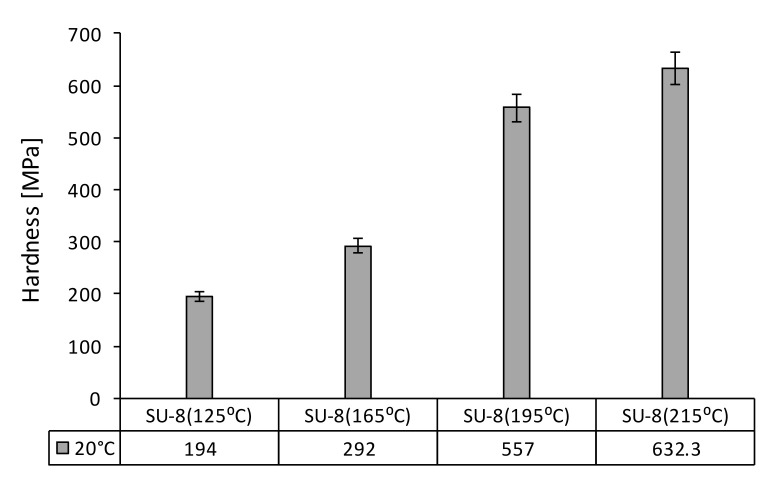
Hardness of SU-8 hard baked at 125 °C, 165 °C, 195 °C, 215 °C tested at 20 °C.

**Figure 6:**
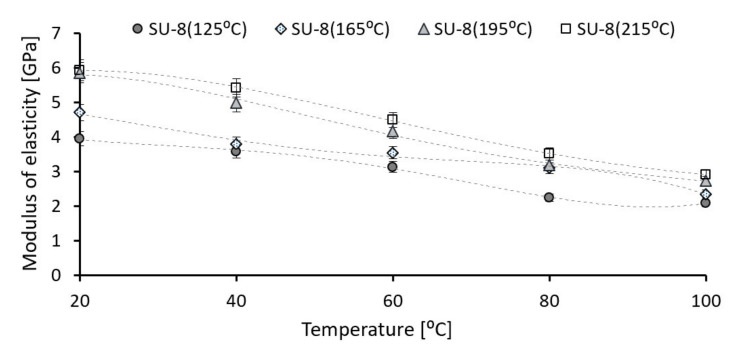
Testing temperature influence on modulus of elasticity of SU-8 hard baked at different temperatures (error amount percentage less to 5%).

**Figure 7:**
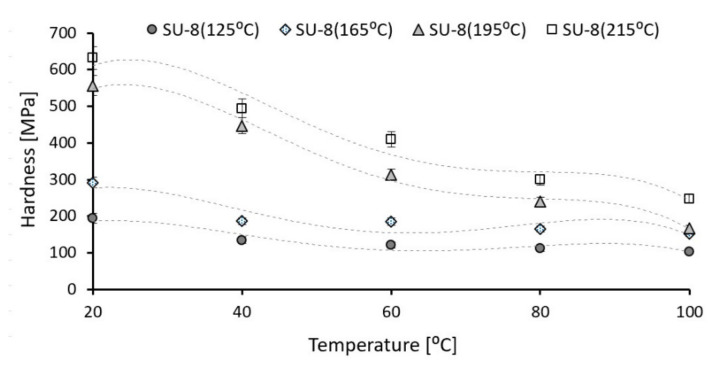
Testing temperature influence on hardness of SU-8 hard baked at different temperatures (error amount percentage less to 5%).

First, the nanoindentation tests were carried out for all investigated SU-8 samples at 20 °C under an indentation force of 50 µN. Figure 4 presents the influence of the hard bake temperature on the modulus of elasticity. The hard bake temperature effect on hardness is presented in Figure 5. The modulus of elasticity and the hardness increase if the hard bake temperature increasing, respectively. Secondly, for each sample the testing temperature is step-by-step increased from 20 °C to 100 °C. Figure 6 presents the effect of testing temperature on the modulus of elasticity of investigated samples. The hardness variation as a function of the testing temperature is presented in Figure 7.

The modulus of elasticity and the hardness decrease for all investigated polymers if the testing temperature increases, respectively. The modulus of elasticity decreases by approximately 47–53% if the operating temperature is increased from 20 °C to 100 °C. The same evolution also is experimentally determined for hardness. The hardness of investigated SU-8 polymers significantly decreases (approximately 46–70%) if the operating temperature increases from 20 °C to 100 °C.

### 3.3. Temperature Effect on Adhesion

One of the operational failure modes (stiction) of MEMS devices with a direct contact between elements is based on the surface adhesion effect. The influence of surfaces roughness, operating temperature, and material properties on the contributions of the adhesion effect must be considered for the tribological reliability issue improvement. The results of experimental tests developed in this paper can be used for the reliability improvement of microdevices with integrated SU-8 as a contact layer in order to prevent adhesion failure mode in terms of roughness, applied contact force and the contact pressure. In the case of grippers application for manipulation, the adhesion effect between SU-8 layers and samples can be influenced by the needed temperature for electro-thermally actuation. The effect of temperature on adhesion effect of SU-8 based on AFM tests is investigated and presented in this section. The tests involve the adhesion force analysis between SU-8 samples and the AFM tip (Si). The operating temperature influence on the adhesion force is experimentally analyzed based on the AFM spectroscopy-in-point. This technique is often used to determine the adhesion between investigated materials and the AFM tip [20,21].

The characteristics of surfaces which are coming in contact, including the roughness parameters, affect the adhesion force [20].

**Figure 8:**
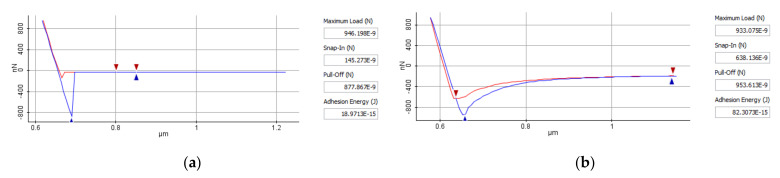
AFM spectroscopy-in-point of SU-8 hard baked at 125 °C, tested at 20 °C (**a**) and 100 °C (**b**).

The spectroscopy-in-point force of AFM curves give information about the direct measurement of tip and samples interaction forces as a function of the distance between them. During the approach, no interactions occur between the tip and the sample surface. As the tip-surface distance becomes sufficiently small, the gradient of the attractive force overcomes the cantilever spring constant and brings the tip into contact with the sample surface (the red line from Figure 8). Further approaching causes a deflection of the cantilever. On the unloading part of the force–displacement curve (the blue line from Figure 8) the deflection of the cantilever decreases as the tip retracts from the sample surface. When the elastic force of the cantilever overcomes the force gradient the tip snaps off from the surface and the cantilever returns to its equilibrium position [29].

The adhesion force (pull-off force) between the AFM tip and investigated samples as well as the snap-in force and adhesion energy are directly provided by the XEI interpretation software. Figure 8 presents the spectroscopy-in-point curves of the SU-8 sample hard baked at 125 °C and tested at 20 °C and 100 °C. The adhesion force between AFM tip and sample is 877.867 nN (Figure 8a) for a testing temperature of 20 °C and it increases to 953.613 nN if the test is performed at 100 °C (Figure 8b). Under the same applied force, the contact area between AFM tip and samples increases if the operating temperature is increased, based on viscoelastic material behavior. Therefore, the adhesion force between AFM tip and investigated SU-8 polymers increases. The same experiments were done for each investigated sample for a testing temperature of: 20 °C, 40 °C, 60 °C, 80 °C,100 °C.

**Figure 9:**
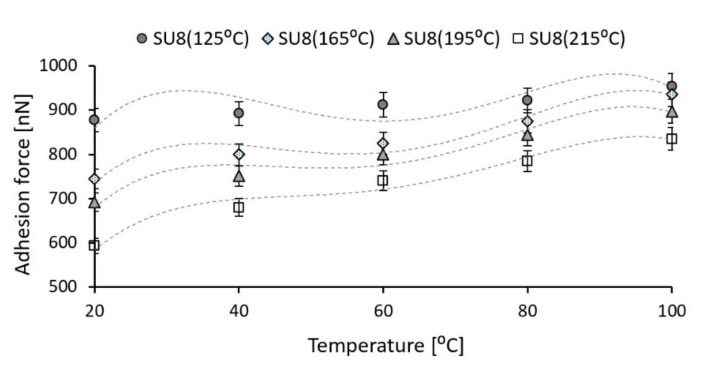
Experimental variation of adhesion force between AFM tip (Si) and SU-8 as a function of the testing temperature (error amount percentage less to 5%).

Figure 9 presents the effect of the testing temperature on the adhesion force between the AFM tip (Si) and the investigated SU-8 polymers hard baked at different temperatures. For all investigated SU-8 polymers, the adhesion force between AFM tip and samples increases as the testing temperature increases, respectively. A thermal gradient of 80 °C applied on investigated materials increase the adhesion force with 8.6% in the case of AFM tip in contact with SU-8 polymer hard baked at 125 °C, 25.5% and 29.7% for the polymers hard baked at 165 °C and 195 °C, and 40.8% for the polymer with 215 °C the hard bake temperature.

### 3.4. Temperature Effect on Friction

In order to fabricate reliable and long-term stability MEMS structures with SU-8 as an integrated layer, attention must be focused on the frictional behavior. Due to the viscoelastic nature of polymer, the frictional behavior of this material is more complicated than the friction of metal.

The friction behavior of SU-8 hard baked at different temperatures was investigated using the lateral mode of AFM. The rotational deflection of AFM probe is optically monitored while the AFM tip (Si) is moved in lateral direction on samples under a controlled normal load. The experimental investigations were performed at different testing temperatures for all investigated samples.

**Figure 10:**
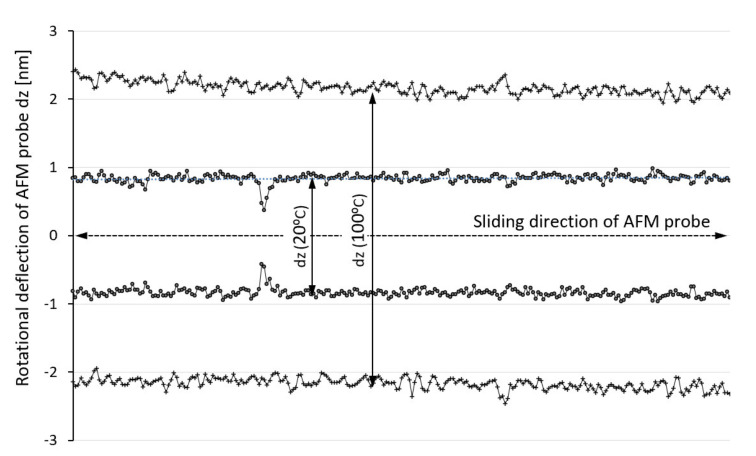
Rotational deflection (dz) of AFM probe during sliding on SU-8 hard baked at 125 °C, tested at 20 °C and 100 °C.

The AFM probe is moved in a lateral direction with a direct contact between the tip and investigated polymers. During lateral movement, the dz rotational deflection of AFM cantilever is experimentally monitored. The rotational deflections of the AFM cantilever are directly proportional with the friction force between the AFM tip and samples. During testing, the temperature was sequentially increased from 20 °C to 100 °C. Figure 10 presents the variation of the torsional deflection (dz) of AFM probe in the case of SU-8 hard baked at 125 °C and tested at 20 °C and 100 °C. Initially, the AFM probe was moved in the lateral direction close to the sample but without any contact with it under different temperature, and the rotational deflection of AFM probe was monitored. No influence was detected of the thermal field on the rotational deflection of AFM probe.

Based on the torsion beam theory, the friction force between AFM probe and investigated polymers can be determined as [29]:

(1) $$F_{f}=\frac{dz\times r\times G\times h^{3}\times b}{l^{2}\times s}$$

where dz is the deflection of AFM probe [nm], r—a coefficient equal to 0.33, G—the shear modulus of the AFM cantilever material, l—the cantilever length, h—the cantilever thickness, b—the cantilever width, s—tip height of the AFM probe.

**Figure 11:**
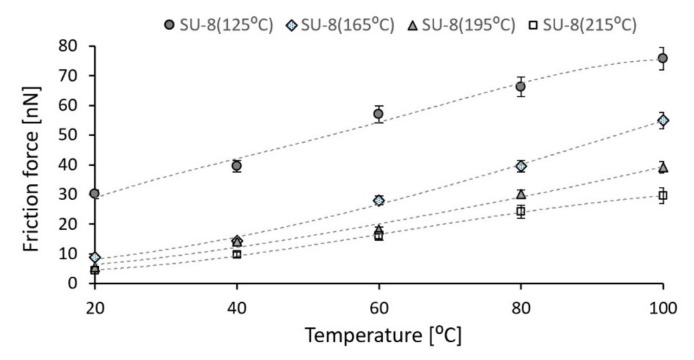
Friction force variation between AFM tip and SU-8 polymers as a function of the testing temperature (error amount percentage less to 5%).

The variations of the friction forces for all investigated polymers are presented in Figure 11. It is known that the contact area has influence on the friction force for polymers [27,28]. Initially, when the testing temperature was smaller, there was a greater contact area between the AFM tip and SU-8 polymers. Under the same applied normal force (300 nN), the contact area increases as the temperature increases, with effects on the friction force.

## 4. Discussion

Due to its low modulus of elasticity and high aspect ratio, the SU-8 has emerged as a structural material in MEMS devices. However, for thermal or electro-thermal applications, it is critical to consider the mechanical and tribological properties variation as a function of temperature. The research work presented in this paper was performed based on the AFM technique for investigation of the mechanical and tribological properties evolution of SU-8 polymer for different processing and operating temperature.

First, the experimental tests were performed at a temperature of 20 °C on SU-8 samples hard baked at 125 °C, 165 °C, 195 °C and 215 °C. The technical SU-8 datasheets recommended a controlled hard bake to further cross-link the SU-8 structures when they will remain as part of the device. The entire process of fabrication should be optimized for the specific application anyway. This step is usually required if the final device or part is subjected to thermal processing during operation. A hard bake or final cure step is added to ensure that SU-8 properties will not change in its use. The SU-8 is a thermal resin and as such its properties can continue to change when exposed to a higher temperature than previously encountered. The technical datasheet provided by MicroChem recommend using a final bake temperature 10 °C higher than the maximum expected device operating temperature. Depending on the degree of cure required, a bake temperature in the range of 150 °C to 250 °C and for a time between 5 and 30 min is typically used.

As the processing temperature increases from 125 °C to 215 °C, the modulus of elasticity increases by 35% and the hardness by approximately 70%. For the same applied force, the adhesion force between AFM tip (Si) and the investigated samples decreases approximately 32% if the hard bake temperature increases from 125 °C to 215 °C. As the hard bake temperature is increasing, the hardness increases and the contact area between the AFM tip and investigated samples decreases under the same applied force, which leads to reducing the adhesion forces. The same effect was observed in the case of the friction force between AFM tip (Si) and investigated samples. For a constant normal force, the friction force significantly decreases (approximately 85%) if the hard bake temperature is increased, the high friction force corresponds to the SU-8 hard baked at 125 °C and the smaller one to the SU-8 hard baked at 215 °C. As the hard bake temperature increases, the hardness of investigated polymers increases, and the AFM tip slips much more easily on the contact surfaces. However, the mechanical and tribological properties of SU-8 are very sensitive to the processing temperature. After being baked at high temperature, due to the increase in the cross-link density, the material becomes harder with the effect on the investigated mechanical and tribological properties.

Secondly, the testing temperature is step-by-step increasing from 20 °C to 100 °C using a thermal controlled stage and the influence of operating temperature on mechanical and tribological properties of SU-8 was analyzed. The mechanical properties as modulus of elasticity and hardness decrease if the testing temperature increases. The modulus of elasticity decreases with 47–53% and the hardness with 46–70%; if the testing temperature increases from 20 °C to 100 °C, the smaller value is determined for the SU-8 hard baked at 125 °C.

Moreover, the investigated tribological properties as adhesion and friction forces increase if the operating temperature increases, respectively. A thermal gradient of 80 °C applied to investigated materials increase the adhesion force with 8.6% for the AFM tip in contact with SU-8 hard baked at 125 °C, with 25.5% and 29.7% for the SU-8 hard baked at 165 °C and 195 °C, and with 40.8% for the SU-8 with the hard bake temperature of 210 °C. The friction forces are modified with 60–86% if the testing temperature increases. The smaller value is determined for the SU-8 hard baked at 125 °C. As the operating temperature increases, the hardness decreases and the area between contact elements increases. This aspect leads to an increase in the adhesion and friction forces between AFM tip and investigated samples.

From the results interpretation, it can be concluded that the SU-8 has viscoelastic behavior dependance on temperature. As a material for many MEMS applications under electro-thermal actuation, the variation of the mechanical and tribological properties as a function of temperature must be considered for the reliability design improvement.

## 5. Conclusions

The effect of processing and operating temperatures on the mechanical and tribological properties of SU-8 polymer were investigated to characterize their behavior in a broad range of temperature.

The hard bake temperature has influence on the mechanical and tribological properties of SU-8 polymer. The effect of hard bake temperature on modulus of elasticity and hardness was observed as well as the hard bake temperature influence on the adhesion and friction forces.

Moreover, the effect of operating temperature on the elastic and viscoelastic material properties of SU-8 hard baked at different temperatures, with influence on the material mechanical and tribological behavior, was analyzed. The operating temperature has influence on the thermal relaxation of investigated materials and on their viscoelastic behavior. The modulus of elasticity and hardness are decreasing, and the adhesion and friction forces are increasing, if the operating temperature increases, respectively.

The results presented in this paper, concerning the evolution of the mechanical and tribological behavior of SU-8 as a function of temperature, are useful for MEMS designers in order to maintain the rigidity of the structure and the output parameters of systems at different operating temperatures.

## Data Availability

Not applicable.

## References

[1] Gelorme J., Cox R., Gutierrez S. (1989). Photoresist Composition and Printed Circuit Boards and Packages Made Therewith.

[2] Feng R., Farris R.J. (2002). Influence of processing conditions on the thermal and mechanical properties of SU8 negative photoresist coatings. J. Micromec. Microeng..

[3] Hammacher J., Fuelle A., Flaemig J., Saupe J., Loechel B., Grimm J. (2008). Stress engineering and mechanical properties of SU-8-layers for mechanical applications. Microsyst. Technol..

[4] Desta Y., Miller H., Goettert J., Stockhofe C., Singh V., Kizilkaya O., Weber W. Deep X-ray lithography of SU-8 Photoresist: Influence of process parameters and conditions on microstructure quality. Proceedings of the Workshop on High Aspect Ratio Microsystem Technologies.

[5] Palacio M., Bhushan B., Ferrell N., Hansford D. (2007). Nanomechanical characterization of polymer beam structures for BioMEMS applications. Sens. Actuators A Phys..

[6] Palacio M., Bhushan B., Ferrell N., Hansford D. (2007). Adhesion properties of polymer/silicon interfaces for biological micro-/nanoelectromechanical systems applications. J. Vac. Sci. Technol. A.

[7] Keller S., Blagoi G., Lillemose M., Haefliger D., Boisen A. (2008). Processing of thin SU-8 films. J. Micromech. Microeng..

[8] Abgrall P., Conedera V., Camon H., Gue A.M., Nguyen N.T. (2007). SU-8 as a structural material for labs-on-chips and microelectromechanical systems. Electrophoresis.

[9] Pustan M., Dudescu C., Birleanu C., Rymuza Z. (2013). Nanomechanical studies and materials characterization of metal/polymer bilayer MEMS cantilevers. Int. J. Mater. Res..

[10] Chung S., Park S. (2013). Effect of temperature on mechanical properties of SU-8 photoresist material. J. Mech. Sci. Tech..

[11] Lee J., Shin H., Kim S., Hong S., Chung J., Park H., Moon J. (2003). Fabrication of atomic force microscope probe with low spring constant using SU-8 photoresist. Jpn. J. Appl. Phys..

[12] Hopcroft M., Kramer T., Kim G., Takashima K., Higo Y., Moore D., Brugger J. (2005). Micromechanical testing of SU-8 cantilever. Fatig. Fract. Eng. Mater. Struct..

[13] Halhouli A.T., Kampen I., Krah T., Buttgenbach S. (2008). Nanoindentation testing of SU-8 photoresist mechanical properties. Microelectron. Eng..

[14] Khoo H., Liu K., Tseng F. (2003). Mechanical strength and interfacial failure analysis of cantilevered SU-8 microposts. J. Micromech. Microeng..

[15] Han L., Joost V. (2009). Determining the Elastic Modulus and Hardness of an Ultrathin Film on a Substrate Using Nanoindentation. J. Mater. Res..

[16] Quaglini V., Dubini P. (2011). Friction of polymers sliding on smooth surfaces. Adv. Tribol..

[17] Petrova D., Sharma D.K., Vacha M., Bonn D., Brouwer A.M., Weber B. (2020). Ageing of Polymer Frictional Interfaces: The Role of Quantity and Quality of Contact. ACS Appl. Mater. Interfaces.

[18] Maver U., Maver T., Peršin Z., Mozetič M., Vesel A., Gaberšček M., Stana-Kleinschek K. (2013). Polymer Characterization with the Atomic Force Microscope. Polym. Sci..

[19] Cristea A., Pustan M., Bîrleanu C., Dudescu C., Floare C.G., Tripon A.M., Banciu H.L. (2021). Mechanical Evaluation of Solvent Casted Poly (3-hydroxybutyrate) Films Derived from the Storage Polyesters Produced by Halomonas elongata DSM 2581T. J. Polym. Environ..

[20] Rusu F., Pustan M., Bîrleanu C., Müller R., Voicu R., Baracu A. (2015). Analysis of the surface effects on adhesion in MEMS structures. App. Surf. Sci..

[21] Grierson D.S., Flater E.E., Carpick R.W. (2005). Accounting for the JKR–DMT transition in adhesion and friction measurements with atomic force microscopy. J. Adhes. Sci. Technol..

[22] Voicu R., Pustan M., Birleanu C., Muller R. An Electro-Thermal Microgripper with Both Closing and Opening In-Plane Movement of the Arms. Proceedings of the 43rd International Semiconductor Conference.

[23] Voicu R., Tibeica C., Müller R., Dinescu A., Pustan M., Birleanu C. (2016). SU-8 microgrippers based on V-shaped electrothermal actuators with implanted heaters. Rom. J. Inf. Sci. Technol..

[24] Somà A., Iamoni S., Voicu R., Müller R., Al-Zandi M.H.M., Wang C. (2018). Design and experimental testing of an electro thermal microgripper for cell manipulation. Microsyst. Technol..

[25] Blagoi G., Keller S., Johansson A., Boisen A., Dufva M. (2008). Functionalization of SU-8 photoresist surfaces with IgG proteins. Appl. Surf. Sci..

[26] Walther F., Heckl W.M., Stark R.W. (2008). Evaluation of nanoscale roughness measurements on a plasma treated SU-8 polymer surface by atomic force microscopy. App. Surf. Sci..

[27] Fischer-Cripps A.C. (2000). A review of analysis methods for sub-micron indentation testing. Vacuum.

[28] Chen J., Bull S.J. (2009). On the factors affecting the critical indenter penetration for measurement of coating hardness. Vacuum.

[29] Pustan M., Muller R., Golinval J.C. (2012). Nanomechanical and nanotribological characterization of microelectromechanical system. J. Optoelectron. Adv. Mater..

